# Microwave Thermal Ablation in an Unusual Case of Malignant and Locally Advanced Rare Tumor of Pancreas in ASA IV Old Male Patient and Literature Review

**DOI:** 10.1155/2018/6064912

**Published:** 2018-04-15

**Authors:** Francesco D'Amico, Michele Finotti, Chiara Di Renzo, Alessio Pasquale, Alessandra Bertacco, Giorgio Caturegli, Gabriel E. Gondolesi, Umberto Cillo

**Affiliations:** ^1^Department of Surgery, Oncology and Gastroenterology, Hepatobiliary Surgery and Liver Transplantation, Padova University, Padova, Italy; ^2^Department of Surgery, Division of Transplantation and Immunology, Yale University, New Haven, CT, USA; ^3^Department of Surgery, Favaloro Foundation, Buenos Aires University, Buenos Aires, Argentina

## Abstract

Pancreatic intraductal papillary-mucinous neoplasm is a rare primary neoplasm of unknown pathogenesis. This kind of tumor represents 0.2–2.7% of all pancreatic cancers and they may proceed to malignant lesions. In this study, we describe a case of pancreatic intraductal papillary-mucinous tumor (4.3 cm) with normal tumoral markers and nuclear atypia. We perform also a systematic review of the literature on MEDLINE and find only one relevant study that used microwave ablation for the palliative treatment of pancreatic tumor. We describe the case of a 70-year-old Caucasian male who was diagnosed with a pancreatic tumor with biliary tree dilatation. The patient underwent computed tomography (CT), percutaneous biopsy, and an endoscopic positioning of prosthesis in the biliary tree. Due to the worsening of jaundice and cholestasis, and considering the severe systemic disease status, palliative surgery with microwave thermoablation in the head of pancreas was performed. No complications were observed. The hospitalization lasted for 11 days after surgery, with normal liver and pancreatic lab tests at discharge. The patient followed a line of chemotherapy for 6 months with a complete response for 8 months. One month after the treatment, a staging CT scan was performed showing the size of the cephalopancreatic lesion had decreased from 43 to 35 mm with signs of complete ablation. The patient had a total response at the imaging of 10 months. One year later, a CT scan follow-up showed progression of the pancreatic disease. The disease remained stable for 18 months. The patient died due to cardiovascular complications with an overall survival of 30 months. Microwave ablation in our case report has been demonstrated to be feasible and safe without complications. It can be used as a phase of multimodality treatment in patients with severe systemic disease status and advanced intraductal papillary-mucinous neoplasm.

## 1. Introduction

Pancreatic intraductal papillary-mucinous neoplasm (IPMN) is caused by proliferation of mucin-producing neoplastic epithelia and characterized by cystic or saccular dilation of the branch duct (BD-IPMN) and/or main duct (MD-IPMN) [1]. The natural behavior of these neoplasms could proceed to malignant lesions. Cells of different oncogenetic potential can be found in the same tumor, representing the natural progression of the disease (adenomas, low- and high-grade dysplasia to in situ carcinoma, to invasive and metastatic carcinoma). Correct distinction between MD-IPMN and BD-IPMN is essential, as the potential malignant evolution of BD is about 25% (ranging from 6% to 46%) while that of MD is 70% [2], and the prevalence of carcinoma in MD can be high (60% to 92%) [1, 3–6].

**Figure 1:**
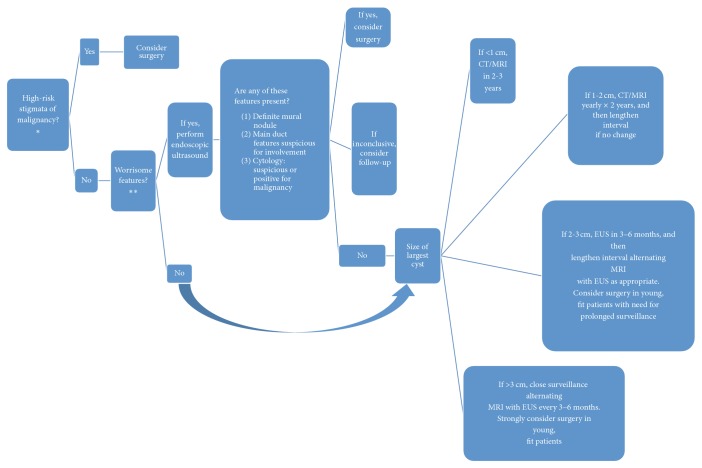
Decisional algorithm for management of IPMN and MCN (international consensus guidelines 2012) [1]. ^*∗*^(A) Obstructive jaundice in a patient with cystic lesion of the head of the pancreas; (B) enhancing solid component within cyst; (C) main pancreatic duct > 10. ^*∗∗*^(A) Cyst > 3 cm; (B) thickened/enhancing cyst walls; (C) main duct size 5–9 mm; (D) nonenhancing mural nodule; (E) abrupt change in caliber of pancreatic duct with distal pancreatic atrophy.

The international consensus guidelines of 2012 allow an evidence-based management of IPMN [1]. Pancreatic resections are indicated for MD-IPMN because of the high rate of malignancy, while the correct management and follow-up of BD-IPMN are debated. These guidelines divide BD-IPMN into two categories: BD-IPMN with “high-risk stigmata” and “worrisome features.” High-risk stigmata include obstructive jaundice, an enhanced solid component, and dilation of the main pancreatic duct (MPD) to a diameter equal to or more than 10 mm. Worrisome features include history of pancreatitis, maximal cyst diameter equal to or greater than 30 mm, a thickened and enhanced cyst wall, MPD diameter of 5–9 mm, nonenhanced mural nodules, abrupt change in the caliber of MPD with distal pancreatic atrophy, and lymphadenopathy. Pancreatectomy is not automatically recommended for patients with worrisome features and follow-up should be considered [7] (Figure 1).

IPMNs are mostly asymptomatic, but can present with long-standing hyperlipasemia, but the most common symptom is acute pancreatitis, due to duct obstruction from mucus or from papillary proliferation. In a recent study at the University of Brescia, Baiocchi et al. [8] followed 40 patients with IPMN during the period from 1992 to 2007 and found less than 50% were symptomatic (principally showing acute pancreatitis at the clinical visit). Since the majority of the patients with IPMN are asymptomatic, the diagnosis is often incidental. Diagnosis of IPMNs, according to the most recent international consensus guidelines of 2012, should be based on imaging studies and typical anatomical and pathological features [1]. The current gold-standard method for the diagnosis of IPMN is Cholangio-Wirsung Magnetic Resonance Imaging (MRI), which appears superior to CT because of better contrast resolution, facilitating recognition of septae, nodules, and duct communications [7]. The specificity of Cholangio-Wirsung MRI in differentiating benign from malignant lesions is increased by using 18-Fludeoxyglucose Positron Emission Tomography (18-FDG-PET, specificity raised from 43% to 100%) [9].

Endoscopic retrograde cholangiopancreatography (ERCP) offers the possibility of performing brushing or biopsy. On the other hand, ERCP may increase the risk of some complications, such as acute pancreatitis. The Surgical Clinic of Brescia [8] evaluated the effects of ERCP and its complications: 50% of the IPMN patients submitted to ERCP developed an iatrogenic pancreatitis. These risk factors explain why the international consensus guidelines for the management of IPMN recommend not to use routine ERCP for sampling of fluid or brushings.

Generally, IPMN can be approached surgically in 90–100% of cases. Pancreatectomy is highly recommended for patients with high-risk stigmata in consideration of the low 5-year survival rates (ranging between 31% and 54%). Patients resected for in situ carcinoma have a median of 5-year survival of 80–90%, 50–70% for invasive carcinoma, and 40%–50% if lymph nodal metastases are present [3]. However, these findings considered cases where pancreatic resection is feasible. On the contrary, we present a case where pancreatic resection for a MD-IPMN evolving to cancer could not be performed due to the performance status of the patient.

## 2. Case Report

We present a case of a 70-year-old Caucasian male admitted to our department in October 2010 due to generalized jaundice. The patient had multiple chronic conditions: hypertensive cardiopathy with double ischemic heart attack, being an active smoker, with previous history of pleurisy with pleural effusion, ischemic stroke five years priorly, and a history of basal cell carcinoma and a treated malignant melanoma in the head.

Due to his comorbidities, according to physical status classification system of the American Society of Anaesthesiologists, the patient was defined as ASA IV (patient with severe systemic disease).

Blood tests showed high direct bilirubin (8.4 mg/dL) associated with an increment of cholestasis indices. Tumoral markers were negative (CEA and Ca 19-9).

**Figure 2:**
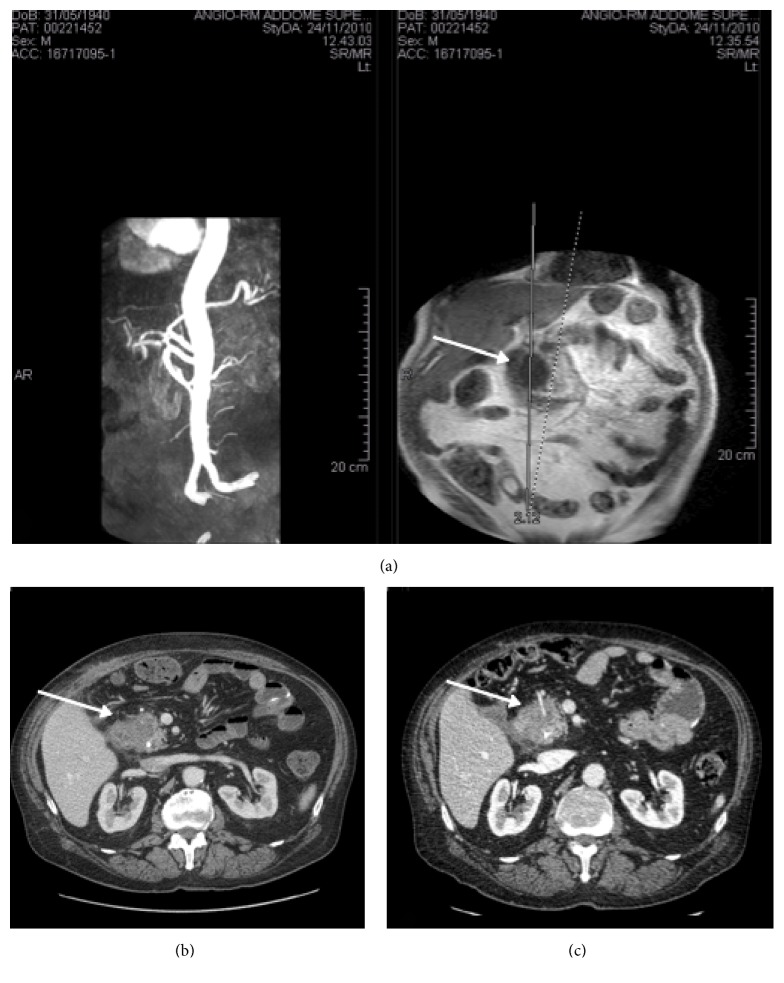
(a) Preoperative staging with an abdominal magnetic resonance plus angiography (MRA) with normal superior and inferior mesenteric and splenic artery. (b) CT scan one month after the treatment. The size of the cephalopancreatic lesion decreased from 43 to 35 mm with sign of complete ablation (no contrast medium uptake). (c) CT scan 10-month follow-up. Progression of the pancreatic disease (40 mm from 35 mm) with suspected initial infiltration of duodenum. The arrows in (a), (b), and (c) refer to the cephalopancreatic lesion.

The patient underwent an abdominal ultrasound followed by a CT scan that showed a hypodense and nonhomogeneous expansive formation (43 mm) in the head of the pancreas with concomitant dilatation of the intra- and extrahepatic biliary tree, without dilatation of Wirsung duct. The lesion was in direct contact of the gastroduodenal artery (GDA) without infiltration. The patient referred to our center completed the staging with an abdominal magnetic resonance plus angiography (MRA) (Figure 2(a)).

Endoscopic retrograde cholangiopancreatography (ERCP) demonstrated a complete obstruction of the intrapancreatic common bile duct. A plastic stent was placed to reduce the bilirubin level and resolve the biliary-tree dilatation.

Percutaneous fine-needle aspiration cytology (FNAC) suggested single or small papillary clusters of epithelial cells with some crowded and hyperchromatic nuclei. These findings were considered compatible with IPMN with high-grade dysplasia.

Due to worsening jaundice and cholestasis, in December 2010 the patient underwent exploratory laparotomy in order to evaluate whether to perform a palliative surgery. The laparoscopic approach due to the severe cardiopathy of the patient was contraindicated.

An exploration of the abdomen was performed, in order to rule out previously undetected metastases. The gastrocolic ligament was divided to access the* bursa omentalis* and a partial Kocher manoeuvre was performed to expose the pancreatic head with the identification of the lesion.

Intraoperative ultrasound studies demonstrated a new evidence of infiltration of the superior mesenteric vein (SMV) and GDA.

Due to the patient's age, his severe systemic disease, and multiple chronic conditions (ASA IV), we decided not to keep on with a duodenocephalopancreatectomy (despite intraoperative signs of rapid progression of the IPMN to cancer) but to perform a palliative surgery and a mini invasive treatment of the tumor with microwave thermoablation (MWA) [19].

An AMICA (apparatus for microwave ablation) system was used. MWA was performed using a 2.45 MHz generator (AMICA-GEN, HS Hospital Service SpA, Aprilia, Italy) delivering energy through a 14- or 16-gauge internally cooled coaxial antenna (AMICA PROBE, HS Hospital Service SpA, Aprilia, Italy), featuring a miniaturized quarter wave impedance transformer (referred to as minichoke) for reflected wave confinement. All procedures were performed under ultrasonographic (US) guidance (Hitachi Hi Vision 6500 convex). The probe was placed directly in the center of the lesion under US guidance, keeping safe margins from the Wirsung and bile ducts.

In order to avoid iatrogenic damage due to the heat produced by the microwave needle, we also placed cold wet gauze over the inferior vena cava and the duodenum was perfused continuously with cold saline solution through a nasogastric tube placed in the second portion of the duodenum.

We performed two cycles of MWA, 60 seconds each, at 20 Watts of power. The effect of the treatment was monitored during the procedure by US. We used a single 3-0 Vicryl stitch to close the microwave needle track in order to prevent possible pancreatic fistula.

In this particular case, the open procedure, compared to a percutaneous approach, allowed us to achieve a complete ablation of the tumor (two cycles with a better volume of necrosis) with high grade of safety.

We completed the procedure with a Roux-Y anastomosis and gastroenteroanastomosis.

The patient had an uneventful recovery without complications and was discharged on the 11th postoperative day with normal liver and pancreatic laboratory tests.

One month after the treatment, a staging CT was performed showing no dilatation of intra- and extrahepatic biliary tree. The size of the cephalopancreatic lesion decreased from 43 to 35 mm with signs of complete ablation (no contrast medium uptake). No lymphadenopathy or metastatic disease was detected (Figure 2(b)).

The patient began a line of chemotherapy from January to July 2011 (12 cycles with Oxaliplatin) and he did not show progression at CT scan for 10 months.

In October 2011, CT scan follow-up (Figure 2(c)) showed progression of the pancreatic disease (40 mm from 35 mm with suspected initial infiltration of duodenum) with single liver metastases (5 mm at the left hepatic lobe).

The disease remained stable for 18 months with normal level of bilirubin but increasing levels of tumor marker (CA 19-9 from 2763, February 2011, up to 7000, November 2011).

The patient died in June 2013 from cardiovascular complications with an overall survival (OS) of 30 months (Table 1).

## 3. Discussion

Our case report described a MD-IPMN, which evolved to a locally advanced pancreatic cancer (LAPC), considered unresectable, due to the performance status of the patient and the stage of the disease (infiltration of the superior mesenteric vein and infiltration of the gastroduodenal artery).

Furthermore, we have to consider that the diagnosis of IPMN is essentially histological after pancreatic resection. In our case report, the preoperative diagnostic tools suggested MD-IPMN, but imaging studies and intraoperative findings suggested a malignant evolution (pancreatic cancer) in a patient considered unresectable for cardiovascular disease.

Recent studies showed an increase of OS in patients affected by pancreatic cancer treated with adjuvant therapy compared to patients treated with surgery alone. The patients who can receive more benefit from the treatment are the ones with higher stage disease [20]. In particular, treatment with gemcitabine seems to be the adjuvant treatment of choice for patients with resected invasive pancreatic cancer [21].

Despite the advances in chemotherapy and chemoradiotherapy regimen, unresectable locally advanced pancreas carcinoma remains a disease with poor prognosis with reported median survival of 9–13 months [22]. Locoregional ablative therapies can be considered possible alternative treatments. Liver tumors are an example where ablative approach has been safely used for a long time. The pancreas, however, entails the risk of complications of associating injuries, especially in relation to duodenum and major vessels.

We performed a systematic literature review using the PubMed and EMBASE databases and the Cochrane Library for studies published in the English language up to December 1, 2016. Only articles that described microwave ablation in unresectable IPMN were initially included. There is no case of MWA used to specifically treat IPMN that could be retrieved; therefore, we increased our search to LAPC.

Table 1 summarizes outcomes, type of ablation, and complications using ablative therapies in premalignant pancreatic disease, as reported in the literature.

Radiofrequency ablation (RFA) is the most common thermal ablation therapy used for LAPC. One study showed a survival rate of 22% with a median follow-up of 12 months [23]. A more recent study showed safety and feasibility of RFA under ultrasonography guidance in 22 patients. The median postablation survival time was 6 months [24]. Experience with MW ablation for unresectable LAPC is limited. The largest case series [25] considered 15 patients in a period between 2004 and 2006 in which all partial necrosis was achieved with no major procedure-related morbidity or mortality using MWA. The longest patient follow-up was 22 months, but the median survival was only 13 months [25].

Our case reports an overall survival after MWA of 30 months. Despite encouraging results, MWA for LAPC as well as its efficacy together with chemoradiotherapy is under investigation, without any conclusive data currently available regarding indication and best ablation protocol for LAPC.

## 4. Conclusions

Most clinical ablative experience for LAPC exists with RFA. There is actually no previous case of microwave thermal ablation use to treat specifically IPMN in rapid progression to cancer. Our case report showed that MWA can be considered a safe and efficacious alternative treatment as a palliative surgery when resection of IPMN is not feasible. However long-term data or randomized controlled trials are required to further characterize this treatment.

## Figures and Tables

**Table 1:** Outcomes, type of ablation, and complications in premalignant pancreatic lesions: review of the literature. CTP: cystic tumors of the pancreas; PNET: primitive neuroectodermal tumor; EUS: endoscopic ultrasonography; CR: complete resolution; NA: not available.

Author	Lesion type	Number	Treatment	Median area of ablation, mm (range)	Outcome	Major complications
Gan et al. 2005 [10]	CTP	25	EUS guided ethanol Lavage	19.4 (6–30)	CR 35%	None

Oh et al. 2008 [11]	CTP	14	EUS guided ethanol Lavage + paclitaxel	25.5 (17–52)	CR 79%	Acute pancreatitis (*n* = 1) Abdominal pain (*n* = 1)

Oh et al. 2009 [12]	CTP	10	EUS guided ethanol Lavage + paclitaxel	29.5 (20–68)	CR 60%	Mild pancreatitis (*n* = 1)

DeWitt et al. 2009 [13]	CTP	42	Randomised double blind: saline versus ethanol	22.4 (10–58)	CR 33%	Abdominal pain (*n* = 5) Pancreatitis (*n* = 1) Cystic bleeding (*n* = 1)

Oh et al. 2011 [14]	CTP	52	EUS guided ethanol Lavage + paclitaxel	31.8 (17–68)	CR 62%	Mild pancreatitis (*n* = 1)

Levy et al. 2012 [15]	PNET	8	EUS guided ethanol Lavage and intraoperative ethanol Lavage	16.6 (8–21)	NA	Peritumoral bleeding (*n* = 3)

Pai et al. 2013 [16]	CTP + PNET	8	EUS guided RFA	38	CR 25%	None

Park et al. 2016 [17]	CTP	91	Ethanol	30 (20–50)	CR 45%	Acute pancreatitis (*n* = 3)

Moyer et al. 2016 [18]	CTP	10	Ethanol or saline plus paclitaxel and gemcitabine	30	CR 75%	Acute pancreatitis (*n* = 1)

## Data Availability

The CT scan is saved in the e-health system of our hospital with the clinical history reported in the case report and is accessible any time if requested by the editor.

## Abbreviations

IPMN: Intraductal papillary-mucinous neoplasm
BD-IPMN: Intraductal papillary-mucinous neoplasm branch duct
MD-IPMN: Intraductal papillary-mucinous neoplasm main duct
ERCP: Endoscopic retrograde cholangiopancreatography
MRI: Magnetic Resonance Imaging
CT: Computed tomography
ASA: American Society of Anaesthesiologists
MRA: Magnetic resonance angiography
FNAC: Fine-needle aspiration cytology
MCN: Mucinous cystic neoplasm
MPD: Main pancreatic duct
MWA: Microwave ablation
LAPC: Locally advanced pancreatic cancer
RFA: Radio frequency ablation
GDA: Gastroduodenal artery
SMV: Superior mesenteric vein
OS: Overall survival.
